# The Swansea RRHiME track: a new curriculum model for embedding rural and remote healthcare into undergraduate medicine programmes

**DOI:** 10.15694/mep.2019.000002.1

**Published:** 2019-01-03

**Authors:** Heledd Iago, Judy McKimm

**Affiliations:** 1 Swansea University

**Keywords:** Rural and remote healthcare, medical students, doctors, medical programme, curriculum, curriculum models, Wales, undergraduate medicine, Welsh language, culture.

## Abstract

This article was migrated. The article was marked as recommended.

Many medical programmes around the world seek to provide experiences for their students in rural and remote health, community and primary care, with a view to encouraging future doctors to practice in under resourced and under-doctored areas of the world. New schools (and satellites of existing schools) have been established in rural and remote areas with a view to recruiting students from those areas who will go on to practice there. In these new schools, and in more traditional programmes, the primary curriculum models in use are either short placements in selected areas (including overseas electives) or immersive longitudinal clerkships, typically in a primary/community setting. A wealth of evidence exists of the success of these approaches, however, for many schools, the curriculum is more fixed and there are limited opportunities for introducing what can be seen as more radical change.

In this paper, we describe a new approach to curriculum design offered by the Graduate Entry Medicine (GEM) programme at Swansea University Medical School which provides a selected group of students within the cohort with a range of opportunities in rural and remote health embedded within the existing curriculum. The Rural and Remote Health in Medical Education (RRHiME) track has been positively evaluated for 7 years and is transferable to a range of curricula and contexts. The longitudinal evaluation has demonstrated that students experience a *prolonged engagement* in rural health environments and/or focused on rural and remote health issues, not in terms of spending time in one context but in obtaining a variety of perspectives about, and deepening their understanding of, the issues, challenges and opportunities encountered in rural and remote settings.

## Introduction

### Background and Context

‘Rurality’ is difficult to describe, and has considerable international variation with regard to the definition. However, it is attributed in most cases to factors pertaining to population, geography or both. For the purpose of this article, populations of 10,000 or less are classed as rural ( The Countryside Agency *et al*., 2004).

**Figure 1.**
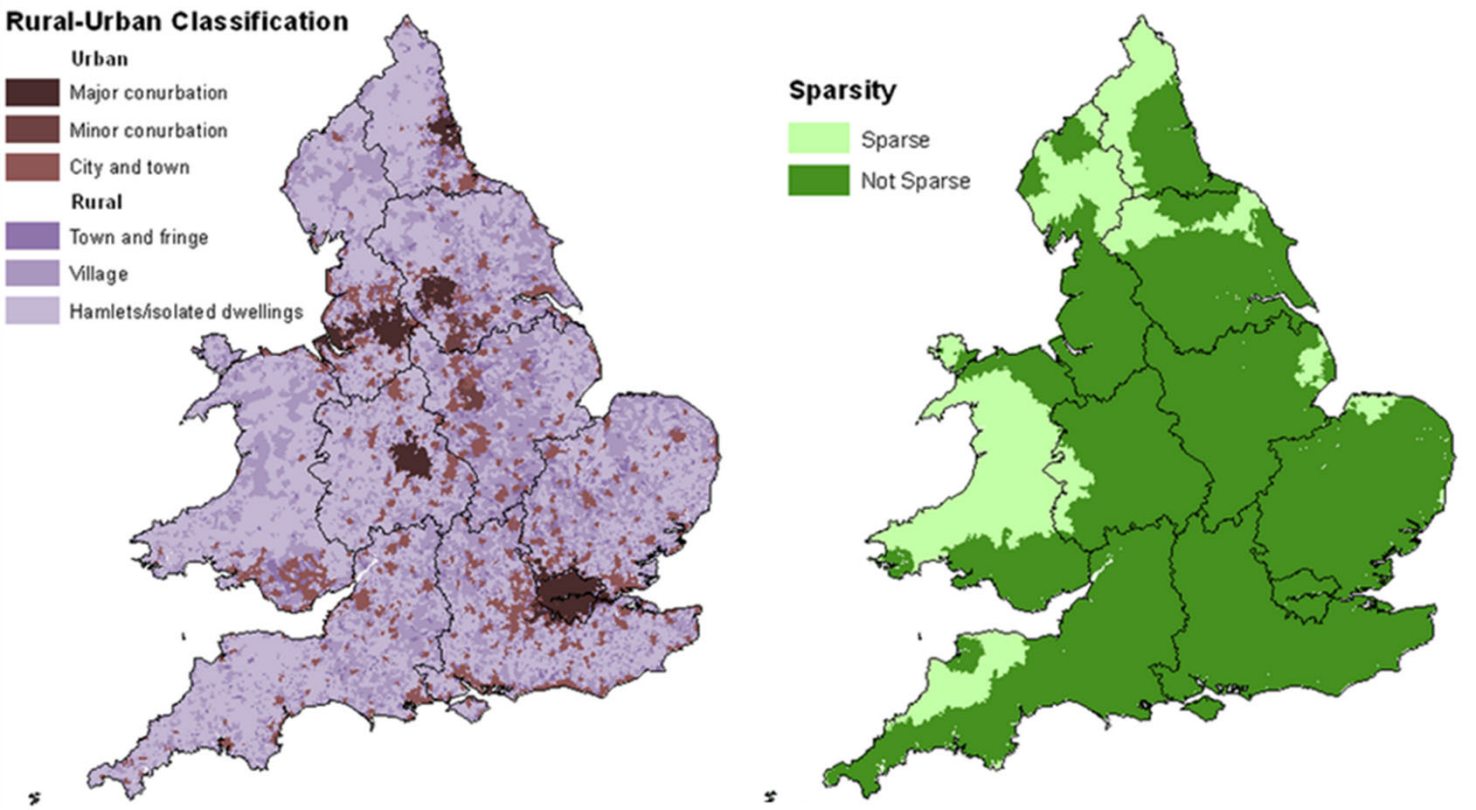
Rural-urban classification 2011 by Output Area.

Source: Office for National Statistics licensed under the Open Government Licence v.3.0. Contains OS data © Crown copyright 2016.

Practice in rural and remote areas can offer varied and interesting benefits to a doctor’s career. However, these rural settings can also often be perceived as particularly challenging clinical environments, contributing to the resulting shortage of qualified doctors (and other health professionals) being recruited to and retained in these areas. This is particularly relevant in Wales, with the clear majority of its landmass classed as both rural and sparse ( Figure 1), and approximately a third of its population living within these rural areas.

**Figure 2.**
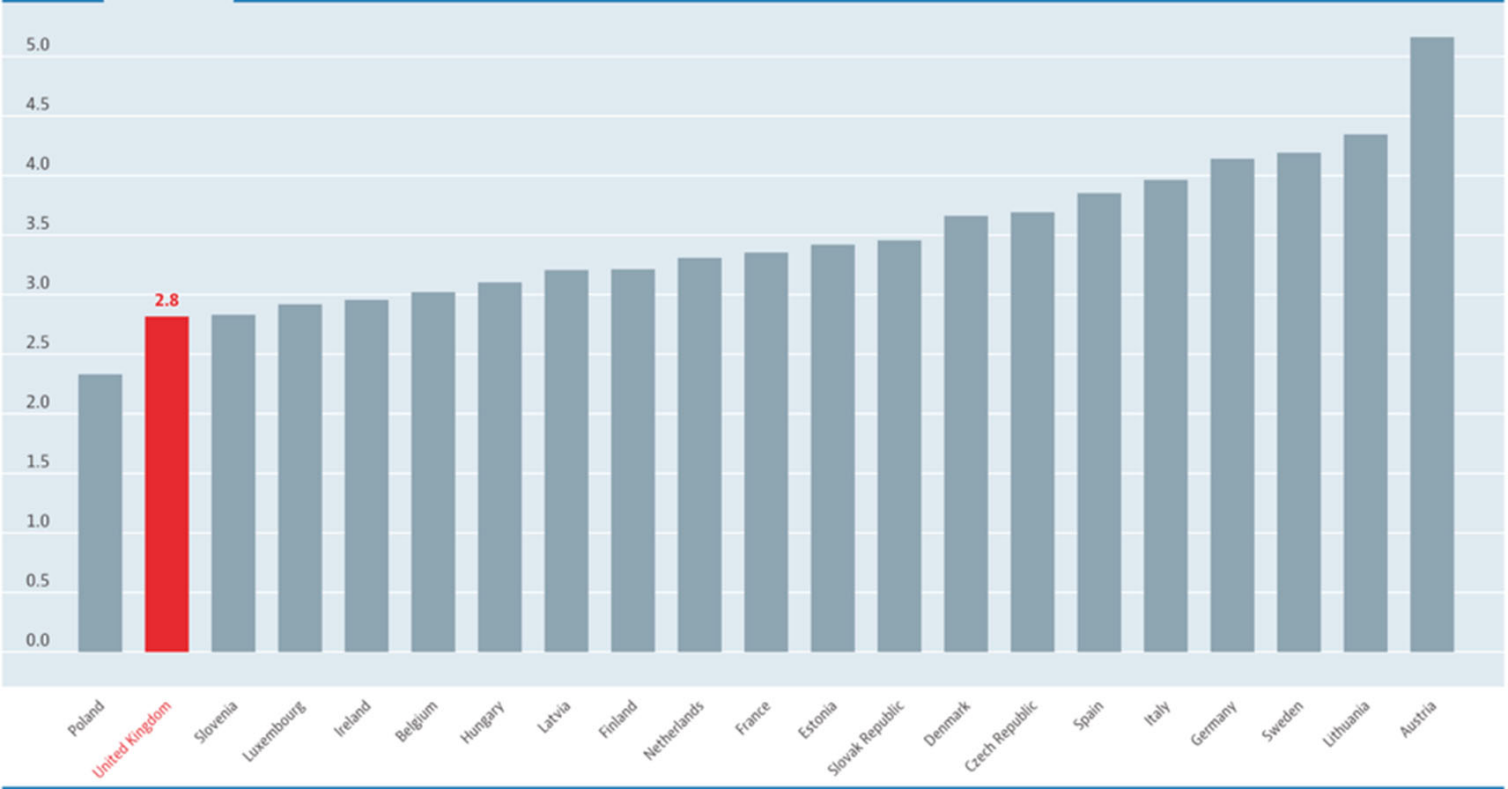
Number of Doctors per 10,000 people in European Countries*.

Wales is suffering from a longstanding and on-going shortage of doctors - now having one of the lowest doctor:patient ratios in Europe ( Figure 2).

(OECD, 2018) *Data not available for all European countries.

International research indicates that early, deep and immersive exposure to clinical experience in a rural healthcare environment may be a contributing factor in facilitating the retention of medics to these rural areas ( Walker *et al*., 2012, Ranmuthugala *et al*., 2007) . This research has been acknowledged and implemented in Wales ( Edwards *et al*., 2015). The *‘Rural Health Plan for improving integrated service delivery across Wales*’ ( Welsh Government, 2009) makes the following recommendations:

“..rural medicine forming part of the medical undergraduate curriculum and the foundation programme..”

“..the location of careers and training posts for medical students and post-graduate levels in rural areas..”

“..the location of career posts has a significant impact on where the doctors choose to live..”

In response to these broader agendas, since 2011, Swansea University Medical School (SUMS) has provided a unique opportunity for its Graduate Entry Medical (GEM) students. A selected number of students each year are given the chance to focus elements of their studies on Rural and Remote Health in Medical Education (RRHiME). The RRHiME track is clearly defined yet fully embedded throughout the whole curriculum ( Table 1), and aims to raise students’ awareness of the benefits and realities of living and working in rural and remote areas. The University’s proximity to many rural areas provides ample and varied opportunities for students to experience consistent and embedded opportunities in a wide range of clinical placements in rural areas, learning about rural services and practices.

## Methods

### The Rural and Remote Health in Medical Education (RRHiME) track

Development of the RRHiME track was based on research from around the world on what curriculum enagagement is most effective in encouraging graduates to work in rural and remote areas (e.g. Worley *et al*., 2000, Tesson *et al*., 2005, Henry, Edwards and Crotty, 2009, Walker *et al*., 2012). All 70 first year students are introduced to RRHiME with a short presentation during induction week. Interested students are invited to apply to join the RRHiME track by writing a 300 word report on their background and interest in rural health, as well as reasons why they would like to join. The reports are read and reviewed by the track lead, and successful applicants are notified via email. There is currently no cap on RRHiME student numbers, and to date, all applications have been accepted. Students stay on the track throughout their four-year course.Since 2011, numbers have steadily increased (from 4 students in 2011 to 14 in 2017) with 45 students (from a total of approximately 280) being on the track in 2017/18.

A RRHiME curriculum was developed, which mapped all activities and placements in the GEM course and considered how a rural and remote ‘lens’ could be applied. RRHiME students follow a negotiated, tailored programme which meets their interests as well as providing prolonged opportunities to work in rural and remote areas and gain appreciation of the specific needs of rural and remote communities. Students are encouraged to engage with, and complete the activities set out in Table 1.

**Table 1.** Curriculum content of RRHiME

GEM Curriculum Element	Description	RRHIME expectation	Examples
Learning Opportunities in the Clinical Setting (LOCS)	Students select 20 single-session clinical experiences across years 1+2 ( Noor, Batra and Byrne, 2011)	Students select up to 10 LOCS relevant to RRHiME	sessions focussing on access, conditions, context, community
Community Based Learning	(CBL) Students attached to a GP surgery for one day, every three weeks, in Y1-3 with a block placement in Y4	CBL during one year of the programme (this may be the one day placements in Y1-3 or the Junior Assistantship in Y4).	At least one placement in a rural or remote location, insight into role of rural GP and specialist health professionals
Student Selected Clinical Projects	Students complete 8 transferable skills projects on the topic of their choice (within curriculum themes)	2-4 student selected component projects with a rural and remote focus	includes poster, oral presentation, literature review, audit
Year-long case studies (Y1-3)	Y1 - Family case study (‘follow’ family through pregnancy and with a new baby) Y2 - Living with a diagnosis (‘follow’ a patient who has had a life changing diagnosis)	RRHiME focus for one case study	Fundamental aspect of the report relevant to RRHiME (e.g. patient’s background, access to care, etc).
Elective	A 6-week student-selected and managed period of study at another institution, in the UK or overseas	Students complete a RRHiME-relevant elective	Often work overseas in R&R setting e.g. Northern Canada, South Africa, Northern Scotland, the Amazon, Pacific islands
Year 4 project	Project in either research, leadership or education	RRHiME-relevant focus	Under a RRHiME supervisor - take a R&R lens on the study e.g. quality or service improvement; education in dispersed settings
Clinical Apprenticeships	5 week clinical placements, students attached to consultant, to ‘learn about being a doctor’. Students complete a total of 9 throughout the 4-year course.	Students complete 2-3 of their apprenticeships in a RRHiME-relevant environment	Allocated to smaller general hospitals, community hospitals, outreach services, integrated care
TOTAL	~30% of total curriculum time		

As seen in Table 1, students are given opportunity to engage with a variety of experiences. For clinical placements in primary and secondary care, clinical placement co-ordinators are sent a list of RRHiME students. These students are preferentially sent to rural areas for their clinical placements wherever possible. Further development includes equipping the students with an evidence-based list, and encouraging them to seek out specific experiences and contexts that have been noted as characteristic of rural healthcare. These include:

1. Community Hospitals
2. The role of the GP as part of the wider community and primary care team
3. Cancer care in a rural setting
4. Palliative and end of life care
5. Telemedicine: clinician to clinician consultation
6. Teleconferencing to support effective Multi-Disciplinary Team working
7. Paramedic first responders
8. Air ambulance
9. Patient pathway involving primary care/ secondary care/ tertiary care and the impact on the patient and wider family members
10. Social/green prescribing and community resilience
11. Welsh language
12. Rural social activity

(Source: Mid-Wales Healthcare Collaborative, www.midwalescollaborative.wales.nhs.uk/home)

Most students undertake their elective overseas, often in remote settings (e.g. Alaska or the Scottish islands) or in rural areas (e.g. in African countries, Canada or Australia). Students work with a RRHiME supervisor on the smaller projects and case studies to identify how these can be angled towards consideration of rural and remote healthcare .

A range of extra curricular elements have also been introduced. Termly University-based meetings are held to support students and provide a forum to discuss opportunities and ideas and share experiences across all four years of the programme. These meetings are held every month or two (depending on student availability due to placement comitments and exam periods). Past activities have included visiting the Air Ambulance Base, Brecon Beacons’ Mountain Rescue speakers, speakers from WONCA Rural and rural GPs, representatives from local Health Boards speaking about health innovations and projects (e.g. telemedicine, integrated health/social care) and students speaking about their elective and other experiences in RRHiME settings. RRHiME therefore links closely with many co-curricular activities that students currently pursue and bring formal opportunities for students engaged in (for example) Wales for Africa projects, Wilderness Medicine, overseas expeditions, Mountain Rescue or the GP Society to graduate with deeper experience in rural and remote medicine. Students are also encouraged to participate and present and conferences and join associations with a focus on rural and remote health.

In 2015, an online RRHiME student portfolio was introduced. This allows the students to record, write and reflect on the specified items in the RRHiME curriculum as well as any RRHiME related experiences in between meetings. The portfolio is accessible through the student’s Blackboard™ site, and can be accessed off-campus. The students are encouraged to use their portfolios as a reflection tool, as well as a record of rural health encounters and experiences that can be printed into a portfolio at the end of their studies.

### Welsh language

For those working in Wales “i *t is important for people working in health, social service and social care to recognise that many people can only communicate their care needs effectively through the medium of Welsh. For many Welsh speakers, being able to use your own language must be seen as a core component of care, not an optional extra.”* ( Welsh Government, 2017). Welsh rural areas often demonstrate an increased percentage of Welsh-speakers within their communities, although the number of Welsh speakers is more evenly distributed throughout Wales than is traditionally perceived.

Welsh language and culture is a core part of the medical programme and ‘Welsh for Medicine’ language lessons are offered to first and second year students. Fluent Welsh speakers can complete parts of their course in Welsh and are encouraged to view their bilingualism as an additional ability that may be exploited to provide a better quality of service to Welsh-speaking patients in clinical settings. Non-Welsh-speakers receive support and guidance to build confidence and familiarity with Welsh language and culture as they train and work in Wales. This approach aims to increase awareness of the linguistic needs and barriers faced by health services world-wide, with recognition and respect for language as a healthcare need not novelty. Due to the correlation of high percentage Welsh speakers in rural areas, RRHiME students are further encouraged to attend the bespoke *Welsh for Medicine* language courses that were developed by the Medical School in collaboration with the Swansea Bay Region Learn Welsh Centre.

## Results

The RRHiME track has been formally evaluated since its introduction in 2011, and although evaluation instruments have been modified over the years, a core set of questions has continued to be asked of the students. To date, four surveys have been carried out at the end of academic sessions 2012/13, 2013/14, 2014/15 and 2015/16. Each survey invites all students on the Track at that time, from across all four years of the GEM course, to respond. Responses are anonymous as student numbers are small, quotes are therefore identified by survey year rather than individuals.

The surveys and responses are clustered under five themes:

### Reasons for joining RRHiME

The main reasons for applying for the RRHiME track can be summed up by this respondent: *“to gain more experience in practising medicine in rural locations. To gain a better understanding the unique challenges faced by those providing healthcare in such settings and the innovative strategies used to overcome them”* (2012/13 survey) *.* Many identified that they had a keen interest in practising in rural health once qualified and wanted to gain more experience of rural Wales and work in local communities to make a more informed career choice.

### About RRHiME: what went well, what could be changed or added and what could be improved?

The RRHiME track has grown and developed since its 2010 launch, and student feedback on the Track from all cohorts is overwhelmingly positive. The way in which the curricular components are designed works very well, enabling students to have a lot of choice in placement location and projects. In response to student feedback, the co-curricular components have evolved into a much more structured experience with formally scheduled meetings which have time for student engagement activities and discussions around RRHiME and more variety of guest speakers with experience in a RRHiME related topic. Students particularly value the way in which RRHiME is networked with other student-led societies and activities and hearing from *‘speakers with a portfolio career and the variety of options open to careers in rural/remote medicine rather than the usual ‘run of the mill’ training in hospitals’* (2016/17 survey). Students very much value the visits to different types of service such as Air Ambulance or Mountain rescue, however these are not always easy to organise as students are dispersed around Wales. Students can have individual placements with such services however.

The online RRHiME portfolio, introduced in 2015, has encouraged students to stay engaged with RRHiME in between meetings, not merely as a box-ticking administrative exercise, but an interactive way to inspire and motivate their interest and development as rural medics, and clearly outline what is expected of them as RRHiMErs, as students felt this was unclear in the early years of the Track.

### Examples of experiences and activities: clinical placements, projects and assessments

Students fully engaged with a range of clinical placements in primary, community and hospital settings and clearly valued the effort made to place them in such varied rural and remote contexts. As well as working in family medicine practices and small general hospitals, they had opportunities to engage in integrated and varied community based and regional services, such as outreach (peripatetic) clinics, homecare, pre-hospital care, small cottage hospitals, nurse and midwife led services, and mountain rescue.

Students were also able to apply a RRHiME lens to formal continuous assessments. Examples included giving an oral or poster presentation or undertaking a literature review on an area that interest has been stimulated on through RRHiME (e.g. on frostbite or Ebola). The longer case studies (Year 1 *Family case study*; Year 2 *Living with a diagnosis* and Year 3/4 *Developing professional practice)* gave rich opportunities for students to gain an in-depth understanding of what life is like for patients and families living in rural communities.Some students struggled with identifying topics and obtaining specific RRHiME supervision and this has been strengthened over the last few years.

### Insights gained about rural and remote health

The RRHiME students more clearly recognise the distinction between urban and rural healthcare needs and delivery. Integral to the RRHiME track is learning that healthcare needs and delivery of services vary greatly according to, and as a consequence of, the population demographics and geography of the area. *“As I am from a rural background myself, I have an interest in how delivery of healthcare differs in rural and remote settings. Learning about how we can adapt our practice to do this is a great opportunity”* (2012/13 survey) *.*

Direct exposure to and experience of rural clinical practice also increases awareness of the challenges and barriers of both being a patient and of healthcare delivery in rural areas. Due to the challenging environments of rural settings, many obstacles need to be overcome in order to try to obtain a degree of consistency and quality of care. Direct insight into the potential challenges faced in rural healthcare has altered and enhanced students’ perspectives with regard to adaptation and innovation of healthcare delivery based on availability of resources. For example, *“on the community based placement, I got to accompany the District Nurse to patients that live too far from the practice. This has allowed me to understand the logistical implications of trying to match staff availability with patient need in remote areas”* (2013/14 survey). Another example is *“I have learned about the risks posed to women giving birth in rural communities and the support and training given to midwives .. I have also learned more about accidents and injuries in rural communities and how these are dealt with”* (2013/14 survey).

Without direct experience in rural settings, the RHHiME students would also not have gained such appreciation of the struggles some patients face in travelling and managing their home lives when secondary and tertiary centres are such long distances from one another (e.g. a mother with twin neonates who had to be cared for in different centres) and of the impact of *“social isolation for elderly patients”* (2014/15 survey). Transport difficulties were mentioned by many as a key issue for patients, particularly for an ageing population.

Overall, students gained great appreciation of the difficulties in recruitment and retention, of the need to use resources more efficiently, and how to treat patients who might not have access to centralised, specialist services, investigations or treatment. It is clear that these students are starting to think differently about pathways of care, integration between health and social care, referral and the role of technologies such as telemedicine and point of care services. Many mentioned that their experiences had given them a new perspective on the role of specialist nurses, GP led services, community hospitals and the way in which healthcare is integrated into the local community. They also demonstrate insight into how to obtain a balance between centralised and devolved, community based services: a big issue in Wales.

From a Welsh language perspective, the RHHiME track raises students’ awareness of the importance of being able to communicate with patients and staff in their preferred language and stimulated many to learn more Welsh.

### Influences of RRHiME on career choice and locality

Direct exposure to and experience of rural clinical practice has a positive effect on potential careers in rural locations ( Laven and Wilkinson, 2003, Barrett, Lipsky and Lutfiyya, 2011, Sen Gupta *et al*., 2014). The RRHiME experience aims to prepare and equip our medical students for clinical practice in many different environments, both in Wales and world-wide. For example, one student commented: *“I intend to return to my native country, the Virgin Islands, to practice and the experiences that I can gain from the RRHiME track will allow me to perform better in my country”* (2012/13 survey) *.* Some students learned that there can be a better life balance working in rural communities and to gain appreciation of the opportunities provided for sports and other recreational pursuits when living and working out of a city.

In each of the surveys, students were asked about the likelihood that they would practice (a) in a rural setting and (b) in Wales. Whilst some (predominantly from the early years of the GEM course) said they were unsure and/or that it was too early to decide, the vast majority of respondents stated that they did intend to practice in a rural setting and that the RRHiME track (specifically the inspiring speakers and the range of clinical experiences) had positively influenced this intention. Regarding intention to practice in Wales, the main impact of the Track is that it reveals the range of interesting and varied opportunities available, about which students had not been aware before, for example *“Wales is such a diverse place to study and work and has so much more to offer than I had previously realised”* (2014/15 survey).

## Conclusions

Through embedding rural and remote health education alongside Welsh language and culture, the Medical School has provided its students with enhanced opportunities to experience medicine in these areas, gaining increased knowledge and awareness of the needs of Welsh rural populations as well as further afield. The Track has clearly raised understanding and insight into the healthcare needs of rural populations. It is anticipated that this will enhance the recruitment and retention of more doctors in the localities that need them most and a long-term evaluation of the RRHiME students’ career destinations is planned.

The evaluations have demonstrated that a Rural and Remote Health Track embedded in the curriculum, supported by a co-ordinator, dedicated clinical placements, specialist supervision for assessments and linkage with student led and extra curricula activities and societies can provide a practical and rewarding alternative R&RH experience to a more immersive model. The model should also be transferable to other medical and health care programmes.

## Take Home Messages

• The RRHiME track in the Graduate Entry Medicine programme at Swansea University Medical School demonstrated that a Rural and Remote Health Track embedded in the curriculum, supported by a co-ordinator, dedicated clinical placements, specialist supervision for assessments, and links with student led and extra curricula activities and societies can provide a practical and rewarding alternative R&RH experience to a more immersive model.
• The model should also be transferable to other medical and health care programmes.

## Notes On Contributors

### Dr Heledd Iago

Heledd is a lecturer in Medical Sciences at Swansea University, a member of the Genome and Structural Bioinformatics research group led by Dr Jonathan Mullins and is director of the Rural and Remote Health in Medical Education track for the Graduate Entry Medicine program.

Having completed a PhD in the North West Cancer Research Institite, scientifically, Heledd’s research interests now include computer modelling of protein structures, studying their interactions, and the implication of these interactions. Alongside this, Heledd has a special interested in language as a core component of effective healthcare delivery, and the recruitment and retention of medics to rural practice in Wales, and has presented on these topics at several conferences.

### Prof Judy McKimm

ORCHID ID: https://orcid.org/0000-0002-8949-5067

Judy’s current role is Director of Strategic Educational Development and Professor of Medical Education in the College of Medicine, Swansea University. From 2011-2014, she was Dean of Medical Education at Swansea and before that worked in New Zealand from 2007-2011, at the University of Auckland and as Pro-Dean, Health and Social Care, Unitec Institute of Technology. Judy initially trained as a nurse and has an academic background in social and health sciences, education and management. She worked at Imperial College London from 1994-2004 and was Director of Undergraduate Medicine from 1998-2004, leading the curriculum development and implementation of a new undergraduate medical programme. In 2004-05, as Higher Education Academy Senior Adviser, she was responsible for developing and implementing the accreditation of professional development programmes and the standards for teachers in HE.

She has worked on over sixty international health workforce, reconstruction and education reform projects for DfID, AusAID, the World Bank and WHO in Central Asia, Portugal, Greece, Bosnia & Herzegovina, Macedonia, Australia and the Pacific. She has been a reviewer and accreditor for the GMC, QAA, the Higher Education Academy and the Academy of Medical Educators for many years. She is programme director for the Leadership Masters at Swansea and Director of ASME’s and AMEE’s international Educational Leadership programmes. She writes and publishes widely on medical education and leadership and runs health professions’ leadership and education courses and workshops internationally. Her most recent books are *Global Health* (with Brian Nicholson and Ann Allen), *Health Care Professionalism at a Glance* (with Jill Thistlethwaite), *Clinical leadership made easy* (with Helen O’Sullivan), *Medical Education at a Glance* (with Jill Thistlethwaite and Kirsty Forrest), the *ABC of Clinical Leadership, 2 ^nd^ edition* (with Tim Swanwick) and *Developing reflective practice* (with Andy Grant and Fiona Murphy).
