# Supplementary material for: The Swansea RRHiME track: a new curriculum model for embedding rural and remote healthcare into undergraduate medicine programmes
Source: MedEdPublish (2016). 2019 Jan 3;8:2. [Version 1] doi: 10.15694/mep.2019.000002.1 (PMC10712474; doi:10.15694/mep.2019.000002.1)
Supplement: Peer review history

# Reviewer report: Reviewer response for version 1

**DOI:** 10.21956/mep.19745.r29607

**Published:** 2019-01-18

**Authors:** John Cookson

**Affiliations:** 1 University of Worcester

This review has been migrated. The reviewer awarded 5 stars out of 5 Thank you; of great interest. We are planning something similar but for the whole cohort so we have to make sure that it meets the aspirations of all our students. In that, the dynamic between 'immersion' in one setting and experience of a number of settings is a crucial one to get right.

Reviewer Expertise:

NA

No decision status is available

# Reviewer report: Reviewer response for version 1

**DOI:** 10.21956/mep.19745.r29609

**Published:** 2019-01-18

**Authors:** Balakrishnan Nair

**Affiliations:** 1 Centre for Medical Professional Development and University of Newcastle

This review has been migrated. The reviewer awarded 5 stars out of 5 Meeting the challenges of rural health issues and providing appropriate health care delivery are universal issues. Rural health training and provision of appropriate and adequate services are vital for the health of the community; but many countries are not meeting this challenge. I believe all medical curricula must have rural component. I find this paper very interesting since it provided a lived-in experience for the students, was well led and learner centred. The increasing number of students enrolling also shows it met the learners’ expectations. Communication training is essential in medical curricula and the students speaking the “same language “would have sent a strong message to the rural communities, that the curriculum planners meant business. There are many examples of successful rural program where students were placed longitudinally in rural locations and learning and teaching existed in a “symbiotic “manner.The evaluation was done properly; the reflective portfolios is a good initiative. It would be very interesting do a follow up study to see whether undergraduate enthusiasm and positive experience met the workforce requirements . Congratulations on this rural health curriculum work and the research

Reviewer Expertise:

NA

No decision status is available

# Reviewer report: Reviewer response for version 1

**DOI:** 10.21956/mep.19745.r29610

**Published:** 2019-01-07

**Authors:** Felix Silwimba

**Affiliations:** 1 University of Lusaka

This review has been migrated. The reviewer awarded 5 stars out of 5 this paper has raised practical approaches applicable to all regions of the world. I was raised in a rural area of a sub Sahara African country . went for medical training in the capital city and went to practice back in the rural areas. this time a teach in the capital city public health but somehow I feel out of place to practice in an urban area. therefore, as a medical educator now I find this paper appealing to my feelings in an emotional way.one would think United Kingdom has no rural remote people, but this paper has clarified the point.

Reviewer Expertise:

NA

No decision status is available

# Reviewer report: Reviewer response for version 1

**DOI:** 10.21956/mep.19745.r29606

**Published:** 2019-01-03

**Authors:** Trevor Gibbs

**Affiliations:** 1 AMEE

This review has been migrated. The reviewer awarded 5 stars out of 5 A very well-written and informative paper that covers not just an interesting development in medical education but one that addresses an important aspect of healthcare in the future and the social accountability of medical schools. I enjoyed reading the paper and learned much from that reading. There were two areas that I was looking to learn a little more from:The language issue. It is appropriate that those thinking of entering a Welsh speaking society should learn Welsh, it was good to see that there seemed to be an opportunity to learn it to different levels, but I wondered what the uptake was from those students who were not Welsh or UK citizens who intended to return home post graduationThe longer-term effects. I am sure that it is the authors' intentions to follow-up on their graduates, but these innovations do depend on their longer-term effects on individuals and systems- I look forward to another paper that demonstrates this.I think that this paper is an essential read for those curriculum planners faced with similar difficulties and and to help this perhaps a more detailed description of the RRHiME curriculum could be shared.

Reviewer Expertise:

NA

No decision status is available

# Reviewer report: Reviewer response for version 1

**DOI:** 10.21956/mep.19745.r29608

**Published:** 2019-01-03

**Authors:** Junichi Tanaka

**Affiliations:** 1 tohoku university

This review has been migrated. The reviewer awarded 5 stars out of 5 It is a very very interesting study. By the way, it is Figure 2, how about thinking that it is not per 10000 people, but the number of doctors per 1,000 population?

Reviewer Expertise:

NA

No decision status is available
